# Atypical B-Cell Acute Lymphoblastic Leukemia with iAMP21 in the Context of Constitutional Ring Chromosome 21: A Case Report and Review of the Genetic Insights

**DOI:** 10.3390/ijms26010357

**Published:** 2025-01-03

**Authors:** José Vicente Gil, Gayane Avetisyan, Sandra de las Heras, Alberto Miralles, María del Cañizo, Ángela Rico, María Eli Valerio, Vanesa Díaz, Paula Piñero, Carmen Orellana, José Cervera, Carolina Fuentes, José María Fernández, Eva Barragán, Esperanza Such, Marta Llop

**Affiliations:** 1 Accredited Research Group on Hematology, Instituto de Investigación Sanitaria la Fe, 46026 Valencia, Spain; 2 Onco-Hematology Unit, Pediatrics Service, Hospital General Universitario de Alicante, 03010 Alicante, Spain; 3 Hematology Diagnostic Unit, Hematology Service, Hospital General Universitario de Alicante, 03010 Alicante, Spain; 4 Genetics Unit, Hospital Universitario y Politécnico la Fe, 46026 Valencia, Spain; 5 Centro de Investigación Biomédica en Red de Cáncer, CIBERONC CB16/12/00284, Instituto de Salud Carlos III, 28029 Madrid, Spain; 6 Onco-Hematology Unit, Pediatrics Service, Hospital Universitario y Politécnico la Fe, 46026 Valencia, Spain; 7 Molecular Biology Unit, Clinical Analysis Service, Hospital Universitario y Politécnico la Fe, 46026 Valencia, Spain; 8 Hematology Diagnostic Unit, Hematology Service, Hospital Universitario y Politécnico la Fe, 46026 Valencia, Spain

**Keywords:** iAMP21, acute lymphoblastic leukemia, ring chromosome, optical genome mapping, next-generation sequencing

## Abstract

Recent studies have demonstrated the association between constitutional ring chromosome 21 (r(21)c) and the development of B-cell acute lymphoblastic leukemia (B-ALL) with intrachromosomal amplification of chromosome 21 (iAMP21). iAMP21 acts as a driver which is often accompanied by secondary alterations that influence disease progression. Here, we report an atypical case of iAMP21 B-ALL with a unique molecular profile in the context of r(21)c. The onset of B-ALL occurred significantly earlier than previously reported in iAMP21-ALL, likely due to the presence of r(21)c. Only scarce cases of iAMP21 with concomitant *PAX5* fusions have been reported. Through an extensive genomic characterization, the novel *WWOX::PAX5* as well as 13q12.2 deletion involving *FLT3* overexpression was found. These findings suggest that r(21)c may induce chromosomal instability on chromosome 21, triggering chromothripsis and leading to iAMP21-ALL. This case provides valuable insights to unravel the complex interplay between germline and somatic genetic alterations in leukemia. Moreover, it underscores the need for thorough genetic evaluation and multidisciplinary management in patients with syndromic presentation, particularly when rare genetic events may contribute to hematologic malignancies.

## 1. Introduction

Constitutional ring chromosomes result from the breakage of both end segments of a chromosome during meiosis or early postzygotic mitosis, leading to a ring structure of the affected chromosome and the development of rare genetic disorders in affected individuals. Among them, constitutional ring chromosome 21 (r(21)c) accounts for approximately 10% of all cases, and the clinical manifestations in carriers range from mild to severe developmental delays, intellectual disabilities, learning difficulties, or distinctive facial features. The association between congenital abnormalities of chromosome 21, other than constitutional trisomy, and acute lymphoblastic leukemia (ALL) is exceedingly rare. However, recent studies have demonstrated the association between Robertsonian translocations involving chromosomes 15 and 21 (rob(15;21)c) and r(21)c with the development of B-cell acute lymphoblastic leukemia (B-ALL) with intrachromosomal amplification of chromosome 21 (iAMP21) [1].

B-ALL with iAMP21 (iAMP21-ALL) is defined by the presence of three or more extra copies of *RUNX1* in an abnormal chromosome 21. This subtype represents an entity acknowledged by the World Health Organization (WHO) and International Consensus (ICC) classifications, affecting around 2% of children with B-cell ALL. The median age of patients is 9 years old, with a higher prevalence in males, and low white blood cell and platelet counts [2]. Several studies have underscored an increased relapse rate in iAMP21-ALL patients treated with standard therapy, although this trend can be mitigated with more intensive treatment regimens [3,4].

The formation of iAMP21 involves a complex mechanism that includes one or more breakage-fusion-bridge (BFB) cycles followed by chromothripsis of chromosome 21. This process results in high genetic complexity with various regions affected by heterogeneous amplifications, inversions, and deletions. Despite this variability, common regions are observed in nearly all patients [5]. Due to its nature, iAMP21 typically acts as a driver event in ALL, accompanied by multiple secondary alterations that influence disease progression. These secondary events include deletions in *ETV6*, *VPREB1*, *CDKN2A/B*, and *RB1*; subclonal fusions like *P2RY8::CRLF2*; and mutations in the RAS signaling pathway [1].

Given the complexity of iAMP21 and the necessity for therapy escalation in these patients, accurate diagnosis is crucial. Here, we report an atypical case of iAMP21-B-ALL in the context of r(21)c, where high-throughput technologies identified several co-occurring genetic lesions that may influence disease progression. These lesions include a novel *PAX5* rearrangement and *FLT3* overexpression, which could serve as potential therapeutic targets. Our results underscore the importance of a comprehensive diagnostic approach to allow for the application of precision medicine in patients with iAMP21-B-ALL.

## 2. Case Report

A 2-year-old female was referred to the emergency department with complaints of oral discomfort caused by a cleft palate. A thorough evaluation of the patient revealed facial anomalies including a flat facial profile, retrognathia, and epicanthus; palpebral fissures; language difficulties; and congenital auricular inter-communication; overall suggesting a 22q deletion syndrome. Genetic studies were subsequently carried out after obtaining informed consent in accordance with the Helsinki Declaration. SNP-Array (Agilent Technologies, Santa Clara, CA, USA), containing more than 40,000 probes along the genome, revealed a terminal 21q22 microdeletion (arr[GRCh37] 21q22.3(43,688,594_48,093,361)x1) affecting at least 55 genes (Figure S1). The karyotype in peripheral blood showed a ring chromosome 21: 46,XX,r(21)c (Supplementary Materials). No significant family history was reported. However, both parents were also analyzed and yielded negative results, confirming the de novo nature of the structural variant.

At the age of 5, the patient was admitted for suspected osteomyelitis involving the left hip and shoulder. Whole blood count showed monocytosis (1.3 × 10^9^/L) and thrombocytopenia (122 × 10^9^/L). Imaging studies revealed resolving edema in the distal femur, signs of septic arthritis, myositis, and a small focus of epiphyseal osteomyelitis in the left shoulder.

Peripheral blood evaluation demonstrated pancytopenia with 12% blast cells exhibiting a B-phenotype. Bone marrow aspiration confirmed 95% of medium-sized lymphoid blasts, a high nucleus/cytoplasm ratio, agranular cytoplasms, round nuclei without nucleoli, and residual elements of erythroid and granulomonocytic series in advanced stages of differentiation. Flow cytometry analysis showed 75% of blasts with immature markers (CD10+, CD19+, CD34+, CD45+/− and CD66c+), confirming the diagnosis of common B-ALL.

Optical genome mapping (OGM) revealed complex chromosomal abnormalities, including chromoplexy affecting chromosomes 9, 16, and 18; partial deletion of chromosome 7p; X chromosome trisomy; partial trisomy of chromosome 1q; deletion of the chromosome 16q distal region; deletion of chromosome 20q; a 13q12.2 deletion juxtaposing *FLT3* and *PAN3*; and chromothripsis of chromosome 21 with amplification of the 21q21.3q22.12 region, consistent with iAMP21 (Supplementary Materials).

Multiple ligation probe-dependent amplification (MLPA) SALSA P335 and P327 (MRC Holland, Amsterdam, NL) detected a heterozygous deletion of *IKZF1* (exons 1–7) and *RUNX1* amplification (5–7 copies), respectively. These results supported the diagnosis of iAMP21-ALL.

**Figure 1:**
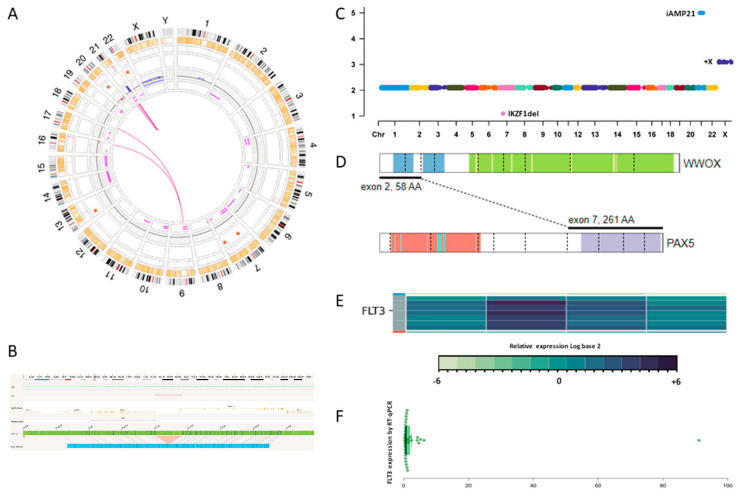
Alterations identified using OGM and NGS. (**A**) Circos plot showing chromoplexy between chromosomes 9, 16, and 18; chromothripsis of chromosome 21; deletions on chromosomes 7, 13, 20, and 22; and trisomy of X. An ALL-directed filter was applied to show clinically relevant variants in this disease. (**B**) 13q12.2 deletion juxtaposing *FLT3* and *PAN3*. (**C**) CNVs identified by NGS (Allseq) and MLPA (P335 and P327). (**D**) Schematic representation of the *WWOX::PAX5* fusion identified using NGS (Archer PanHeme). (**E**) Relative expression of *FLT3* quantified using NGS; the second column shows patient’s expression, while the first, third, and fourth columns belong to healthy controls. (**F**) Relative expression (>90-fold) of *FLT3* compared to 30 healthy controls quantified using RT-qPCR.

Next-generation sequencing (NGS; Allseq [6] and PanHeme (ArcherDx, Inc., Boulder, CO, USA)) revealed additional pathogenic variants: *NRAS* p.Gly12Ser (VAF 10.2%), *FLT3* p.Ile836_Met837insSer (VAF 5.8%), *FLT3* overexpression, and the *WWOX::PAX5* fusion. The latter two alterations were further confirmed using RT-PCR (Figure 1; Supplementary Materials).

Treatment was initiated according to the SEHOP-PETHEMA 2013 protocol, comprising prednisone (60 mg/m^2^/day), triple intrathecal (methotrexate 12 mg, hydrocortisone 20 mg and cytarabine 30 mg), vincristine (1.5 mg/m^2^/day), daunorubicin (1.5 mg/m^2^/day), and asparaginase (10,000 U/m^2^/day). Minimal residual disease (MRD) was positive at day +15 (68%), reassigning the patient to the high-risk group. Currently, 3 months after diagnosis, the patient remains in complete remission.

## 3. Discussion

Chromosome 21 alterations are observed in ~60% of pediatric B-ALL, with certain constitutional alterations, such as Down syndrome or Robertsonian translocations (i.e., rob(15;21)(q10;q10)c), showing an up to ~2700-fold increased risk of developing iAMP21-ALL compared with the general population [5,7]. The presence of r(21)c is also likely to increase the risk of developing leukemia [8]. However, the incidence of this rare abnormality remains uncertain, making it difficult to estimate its impact on leukemogenesis. The presented case further supports the potential relationship between r(21)c and the development of ALL.

iAMP21 is predominantly observed in older children or adolescents, as reported by Harrison et al. [9]. This contrasts with the median age for other childhood ALL subtypes, which generally occur between 2 and 5 years. Nevertheless, it is important to note that early onset of ALL is more common in children with chromosome 21 abnormalities [10]. Notably, in this case, the development of ALL also occurred significantly earlier than previously reported in iAMP21-ALL, likely due to the presence of r(21)c.

Overall, these results support the association between r(21)c and the development of iAMP21-ALL. However, further research involving large cohorts and functional experiments are needed to demonstrate a causative relationship and the molecular underlying mechanism.

iAMP21 is typically defined as a primary oncogenic driver, often accompanied by secondary events. The most frequent concomitant alterations are those affecting the RAS pathway, as observed in the present case. However, the co-occurrence of gene fusions is exceptionally rare. Only a few cases with the coexistence of *ETV6::RUNX1*, *P2RY8::CRLF2*, and *IGH::MYC* have been reported, predominantly at subclonal levels [11].

To the best of our knowledge, cases of iAMP21 with concomitant *PAX5* fusions are scarce. *PAX5* fusions occur in approximately 2.5% of pediatric B-ALL patients and typically result in chimeric genes encoding proteins that retain the DNA-binding paired box domain (exons 1–4) of *PAX5*. These chimeric proteins act as dominant-negative inhibitors, interfering with the activities of wild-type *PAX5* [12]. In contrast to the previously described fusions, we have identified the novel *WWOX::PAX5* transcript, which lacks the paired box DNA-binding domain. The *WWOX* gene (WW domain-containing oxidoreductase) is located in the fragile region FRA16D on chromosome 16q23.3–24.1, an area prone to genetic rearrangements in different types of cancer. Fusions involving this gene seem to disrupt its tumor suppressor function, contributing to oncogenesis [13]. Further studies are needed to elucidate the functional role of the *WWOX::PAX5* fusion in leukemogenesis and its clinical impact.

High levels of *FLT3* have been reported in various ALL subtypes, including high hyperdiploidy and *KMT2A-r* or *ZNF384-r* ALL. Recently, Yang et al. [14] reported that 13q12.2 deletions lead to *FLT3* overexpression in two cases of iAMP21-ALL. Our study corroborates these findings, strongly suggesting that 13q12.2 deletions play a significant role in leukemogenesis and may be particularly enriched in iAMP21-ALL. Interestingly, while *FLT3* mutations are sufficient to induce gene overexpression, their concurrence with 13q12.2 deletions results in a significantly higher expression that may contribute to the aggressive nature of iAMP21-ALL. Randomized studies with FLT3 inhibitors have already demonstrated clinical benefits in infant *KMT2A-r* ALL [15]. More recently, preclinical studies have shown how FLT3 and RAS signaling inhibitors could offer improved treatment options for pediatric iAMP21-ALL patients [16]. These therapeutic approaches could, in general, improve the survival of ALL patients, as the RAS pathway is mutated in over 25% of cases and associated with reduced outcomes and therapy resistance. Therefore, investigating the efficacy of FLT3 and RAS pathway inhibitors in these patients, who often require intensive treatment due to their high-risk status, could potentially improve outcomes. Such targeted therapies may offer a more effective and less toxic alternative to conventional treatments, highlighting the importance of personalized medicine in high-risk ALL subtypes.

## 4. Conclusions

We report an atypical B-ALL patient with a unique molecular profile in a background of r(21)c. These findings suggest that r(21)c may induce chromosomal instability on chromosome 21, triggering chromothripsis and leading to iAMP21-ALL. This case provides valuable insights to unravel the complex interplay between germline and somatic genetic alterations in leukemia. Moreover, it underscores the need for thorough genetic evaluation and multidisciplinary management in patients with syndromic presentation, particularly when rare genetic events may contribute to hematologic malignancies.

## Data Availability

Data are available under reasonable request to llop_margar@gva.es or barragan_eva@gva.es.

## Supplementary Materials

The following supporting information can be downloaded at: https://www.mdpi.com/article/10.3390/ijms26010357/s1.
