# Supplementary material for: Atypical B-Cell Acute Lymphoblastic Leukemia with iAMP21 in the Context of Constitutional Ring Chromosome 21: A Case Report and Review of the Genetic Insights
Source: Int J Mol Sci. 2025 Jan 3;26(1):357. doi: 10.3390/ijms26010357 (PMC11719716; doi:10.3390/ijms26010357)
Supplement: Supplementary file 1 [file ijms-26-00357-s001.zip › ijms-3363163-supplementary.pdf]

Atypical B-cell Acute Lymphoblastic Leukemia with iAMP21 in the Context of Constitutional Ring Chromosome 21: A Case Report and Review of the Genetic Insights

Supplementary methods

Patient and samples

Samples were collected from bone marrow (BM) aspirates at the time of diagnosis. The patient provided informed consent in accordance with protocols approved by the Hospital General Universitario Dr. Balmis (Alicante, Spain) and Hospital Universitari i Politènic La Fe (Valencia, Spain), and in compliance with the Declaration of Helsinki.

SNP-Array

Affymetrix CytoScan HD arrays (Affymetrix, Santa Clara, CA) were used in accordance with the manufacturer's guidelines. Copy number alterations (CNA) and copy-neutral loss of heterozygosity (CN-LOH) were examined using the Chromosome Analysis Suite (ChAS) software version 4.2.0.80 supplied by Affymetrix.

Chromosome banding analysis

Karyotyping (G-banding) was performed on a peripheral blood (PB) sample using standard techniques.

Morphology and immunophenotype

BM aspirate smears were stained with May–Grünwald–Giemsa according to standard laboratory protocols.

Immunophenotyping was performed by flow cytometry on BM samples according to standardized procedures. Antibodies used included anti-CD45, CD19, CD10, CD20, CD34, CD81, CD66c, CD58, CD33, TdT, CD13, CD9, CD123, CD21, CD15, CD65, NG2, TSLP, CD25 (from Beckman Coulter, CA, USA), and IgM, kappa/lambda (from Dako, CA, USA).

Optical genome mapping

Ultra-high-molecular weight (UHMW) DNA was extracted from frozen BM according to the manufacturer’s instructions (Bionano Genomics, San Diego, CA, USA). The WBCs were lysed, digested with proteinase K, and treated with phenylmethylsulfonyl fluoride (PMSF) to obtain genomic DNA. 750 ng of DNA were labeled with a DLE-1 enzyme and kept at room temperatura overnight. Labeled DNA was linearized in nanochannel arrays on a Saphyr chip® and imaged using the Saphyr® Instrument. Sample analysis was conducted using Bionano Access software.

Next Generation Sequencing

Next generation sequencing (NGS) was performed using Allseq (custom panel, Ion Torrent; ThermoFisher Scientific) and Archer PanHeme kit (ArcherDX, Inc, Boulder, USA). Allseq allows the detection of several point mutations, indels, copy number variations, fusions and gene expression relevant to ALL. DNA and RNA libraries were automatically generated using the Ion Chef and sequenced on the Ion S5 sequencer (Thermo Fisher Scientific). The

Archer® PanHeme® Kit targets 199 genes commonly altered in ALL. This panel uses the Anchored Multiplex PCR (AMP), which allows the identification of fusions with known and unknown partners. Library generation was performed manually using 200 ng of RNA according to manufacturer's instructions. Sequencing was carried out on a Ion S5 sequencer and results were analysed on the Archer Analysis Software 6.0 (ArcherDX).

Supplementary data

SNP-Array

Genes affected (OMIM) by arr[GRCh37] 21q22.3(43,688,594_48,093,361)x1 were: *ABCG*1 (603076), *TFF3* (600633), *TFF2* (182590), *TFF1* (113710), *TMPRSS3* (605511), *UBASH3A* (605736), *RSPH1* (609314), *SLC37A1* (608094), *PDE9A* (602973), *WDR4* (605924), *NDUFV3* (602184), *PKNOX1* (602100), *CBS* (613381), *U2AF1* (191317), *CRYAA* (123580), *SIK1* (605705), *HSF2BP* (604554), *RRP1B* (610654), *PDXK* (179020), *CSTB* (601145), *RRP1* (610653), *AGPAT3* (614794), *TRAPPC10* (602103), *PWP2* (601475), *GATD3A* (601659), *ICOSLG* (605717), *DNMT3L* (606588), *AIRE* (607358), *PFKL* (171860), *CFAP410* (603191), *TRPM2* (603749), *LRRC3* (617620), *TSPEAR* (612920), *UBE2G2* (603124), *SUMO3* (602231), *PTTG1IP* (603784), *ITGB2* (600065), *LINC00163* (610259), *PICSAR* (617191), *ADARB1* (601218), *POFUT2* (610249), *COL18A1* (120328), *SLC19A1* (600424), *PCBP3* (608502), *COL6A1* (120220), *COL6A2* (120240), *FTCD* (606806), *SPATC1L* (612412), *LSS* (600909), *MCM3AP* (603294), *YBEY* (617461), *PCNT* (605925), *DIP2A* (607711), *S100B* (176990), *PRMT2* (601961).

Optical genome mapping

Complete OGM formula at diagnosis was ogm[GRCh38] (X)x3,1q21.1q44(143278152_248943333)x3,7p12.2(50348483_50399656)x1,t(16;9;18;20)(q23.1;p13.2;p11.21;q11.23),20q11.21q13.33(32497427_64333718)x1,21q21.3q22.12(30123231_35493059)amp.

Supplementary Figures

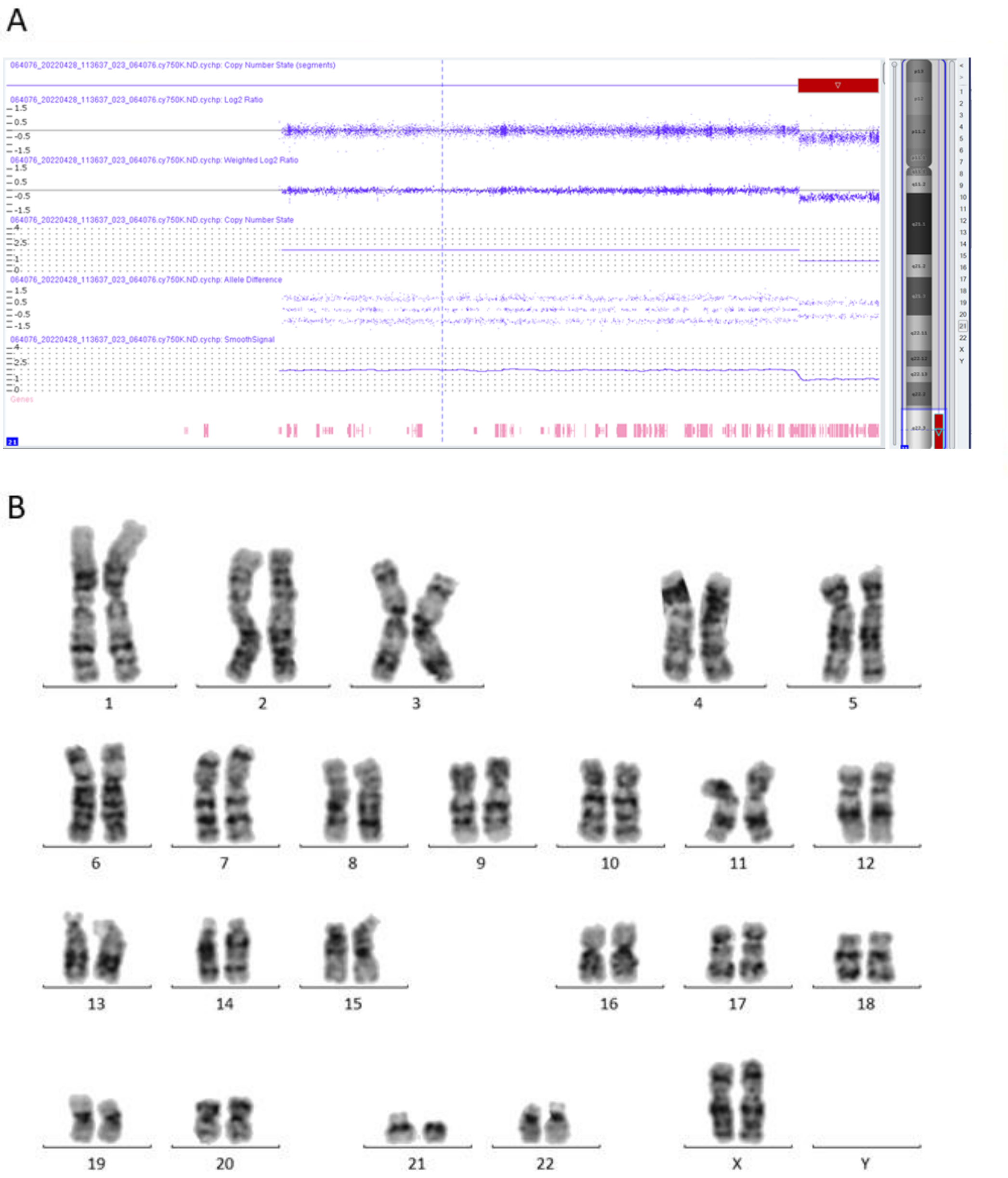

Figure S1. Cytogenetic studies of peripheral blood showing a r(21)c. A) SNP-Array profile showing copy number variation in the terminal region of chromosome 21. The red bar on the right side highlights the deleted area. B) Karyotype analysis with chromosome 21 exhibiting an abnormal ring structure.
